# Leadership through a gender lens: Disparities in Dental Research

**DOI:** 10.1590/0103-644020230555959

**Published:** 2023-12-22

**Authors:** Luisa Gatti-Reis, Flávio Freitas Mattos, Isabela Almeida Pordeus, Paulo Antônio Martins-Júnior, Danielle Carvalho de Oliveira Coutinho, Matheus França Perazzo, Saul Martins Paiva

**Affiliations:** 1 Department of Paediatric Dentistry, Federal University of Minas Gerais - 6627 Av. Antônio Carlos, Belo Horizonte, 31270-901, Minas Gerais, Brazil.; 2 Department of Social and Preventive Dentistry, Federal University of Minas Gerais- 6627 Av. Antônio Carlos, Belo Horizonte, 31270-901, Minas Gerais, Brazil.; 3 Institute of Biological Sciences, Federal University of Minas Gerais- 6627 Av. Antônio Carlos, Belo Horizonte, 31270-901, Minas Gerais, Brazil.; 4 Department of Dental Public Health, Universidade Federal de Goiás - Av. Universitária, s/n.º - St. Leste Universitário, Goiânia, Goiás, Brazil.

**Keywords:** dentistry, bibliometric, leadership, gender equity, sexism

## Abstract

This study aimed to analyze the 100 most-cited papers in Dentistry, with a focus on female leadership in dental research. Papers were retrieved from the Web of Science Core Collection (WoS- CC) in the category ‘Dentistry, Oral Surgery & Medicine’. Gender was assessed through WoS-CC, Scopus, ResearchGate, social media, institutional websites, and software that assigns gender according to first names (https://genderapi.io). Characteristics of authors in leadership roles were retrieved, such as affiliation, publication history, citations, H factor, and i500. The 100 most-cited papers in Dentistry were authored by 394 researchers, 326 (82.7%) men, and 68 (17.3%) women - there were 4.8 male authors for each female. Among the lead authors, there were 11.3 males for each female. Among female senior authors, there were 7 males for each female. Among lead/senior authors of the 100 most-cited papers (first and last authors, respectively), 18 were women. There was an increase in the participation of women in the top cited papers regardless of authorship role across the six decades, with a peak of two female authors in the first decade of the 21^st^ century. For female authors in leadership roles, their publication history shows the time between their first and last papers in WoS-CC ranged from 4 to 42 years for lead authors and 1 to 39 years for senior authors. Women were found to be largely underrepresented as leaders of the 100 most-cited papers, highlighting pervasive gender inequalities in dental research publications.

## Resumo

Objetivou-se analisar os 100 artigos mais citados em Odontologia, com foco na liderança feminina na pesquisa odontológica. Os artigos foram identificados utilizando-se a base de dados Web of Science Core Collection (WoS-CC) na categoria ‘Dentistry, Oral Surgery & Medicine’. O gênero dos autores foi avaliado por meio do WoS-CC, Scopus, ResearchGate, mídias sociais, sites institucionais e um software que atribui gênero de acordo com o primeiro nome (https://genderapi.io). As características dos autores em papéis de liderança foram identificadas, como afiliação, histórico de publicação, número de citações, fator H e i500. Os 100 trabalhos mais citados em Odontologia foram de autoria de 394 pesquisadores, sendo 326 (82,7%) homens e 68 (17,3%) mulheres - foram 4,8 autores homens para cada mulher. Entre os primeiros autores, havia 11,3 homens para cada mulher. Entre os últimos autores, havia 7 homens para cada mulher. Entre os primeiros/últimos autores dos 100 artigos mais citados, 18 eram mulheres. Houve um aumento na participação de mulheres nos artigos mais citados, independentemente do papel da autoria ao longo das seis décadas, com um pico de duas autoras na primeira década do século XXI. Para autoras em papéis de liderança, seu histórico de publicação mostra que o tempo entre o primeiro e o último artigo no WoS-CC variou de 4 a 42 anos para primeiro autor principais e de 1 a 39 anos para último autor. Verificou-se que as mulheres estão sub-representadas como líderes dos 100 artigos mais citados, destacando-se desigualdades de gênero generalizadas nas publicações em pesquisa odontológica.

## Introduction

Gender stereotypes can be diverse, establishing societal values and expectations for women as family-oriented caregivers^1^, and determining their cognitive ability^2^. Evidence shows that in 6- and 7-year-old children, the recognition of the stereotypes of "brilliance" and "genius" as characteristics of their gender is more likely to occur in boys than girls^2^. It may have lasting impacts throughout life, affecting career choice and progression^1^ ^,^ ^2^. In addition, it may generate conscious or unconscious individual bias of lack of impartiality in subjective assessments^3^, in men and women alike^4^.

Current research on the gender gap in science points to disadvantage, not merit or cognitive ability, as responsible for the observed power asymmetries^3^. In Dentistry, despite a marked increase in the participation of women in dental schools in recent years^5^, it is noteworthy that gender inequalities in dental science abound^4^ ^,^ ^5^ ^,^ ^6^ ^,^ ^7^ ^,^ ^8^ ^,^ ^9^. They have been reported in publications^8^, in speakers at dental conferences^9^, and leadership positions across different roles and domains^6^ ^,^ ^7^.

In science, bibliometric analysis can be used to identify research topics and highlight prolific authors, journals, and institutions^10^. One measure of the scientific merit of a paper is the number of citations it receives over time^11^, which indicates its influence^12^.

It can be inferred that authors of top-cited papers occupy a position of leadership, relevance, influence, and prestige in a field. However, little is known about female dental researchers’ representation among them. As a measure of leadership diversity in dental research, the representation of women authors in the most-cited papers in Dentistry needs to be further explored. This paper aimed to assess women as lead, intermediate, and senior authors of top-cited articles in dental research, by characterizing their authorship contribution to the 100 most-cited papers in Dentistry, according to Web of Science Core Collection (WoS-CC).

## Material and methods

This study was carried out using secondary data and therefore was exempted from review by an Ethics Committee.

### Study Design

A bibliometric study was carried out on July 23, 2021, to identify and analyze female leadership among the 100 most-cited papers in dental research through paper authorship. A comprehensive search was conducted at Clarivate Analytics WoS-CC database to retrieve all papers listed in the category “Dentistry, Oral Surgery & Medicine”, according to previously reported methods^12^. No restrictions on language or year of publication were applied.

The papers were organized in descending citation count according to WoS-CC. Two researchers selected the papers and conference papers were excluded. The selection stopped at the 100^th^ most-cited paper which was ranked in descending order of citations. In the case of a draw, the position of a paper in the list was based on the highest citation density (citations/years since publication).

### Variables of interest

The following data were extracted from each of the 100 most-cited papers: authors’ country of institutional affiliation, study design (nonsystematic review, systematic review, laboratory study, non-randomized clinical trial, randomized clinical trial, cross-sectional study, validation study, case/series report, cohort study, and case-control study), study subject, year of publication, number of authors per publication, lead author’s gender, intermediate authors’ gender, senior author’s gender. In this study, gender was assessed as a binary variable, male/female.

According to the Committee on Publication Ethics, the expression “lead author” may assume different meanings across disciplines^13^. It may refer to the first, the most senior, or the last author of a paper^13^. In this study, the term “lead author” was used as a proxy for the first author, as previously used in studies in the fields of medicine, pharmacy, and dentistry^14^. The last authors of the papers were identified as “senior authors”.

The following data were extracted and calculated from WoS-CC for each female lead and senior author included in the 100 lists: country of institutional affiliation, the total number of papers, total citations, H factor, i500, year of publication of first/last paper in the 100 lists, and percentages as first/last author in the database. Only one first author per paper was identified and obtained from WoS metadata. For single-authored papers, the researcher was identified as the first author. The authors’ country of institutional affiliation was obtained from the Scopus database. The citation density of each selected paper was calculated as the total number of citations divided by the number of years spanning between the date of publication and the year 2020. Citation density was categorized in descending order according to quintile: 1) From 913.00 to 76.32; 2) from 74.93 to 55.13; 3) from 52.64 to 33.52, 4) from 31.68 to 21.38, and 5) from 21.11 to 12.40.

The assignment of authors’ gender was carried out individually. The authors’ gender was identified by their first names, obtained from WoS, PubMed, and Scopus. For authors whose gender could not be immediately identified by their first names, the software GenderAPI® (version 3.14) was used. At the time of the study, the software held over 4.000.000 names from 188 countries and calculated the probability of each name being masculine or feminine. The probability of 85% was used as a cutoff point, as already found in the literature.^8^ For authors who scored below this mark, a manual search strategy was carried out and extensively searched: the website of their academic institution affiliation, professional websites (ResearchGate, LinkedIn), social media (Twitter, Facebook), and individual author’s curriculum vitae. After this search, when it remained impossible to determine the authors’ gender, the papers’ corresponding authors were emailed for assistance. In the end, authors whose gender remained unidentified were tagged as “not identified”. The gender identification process was double-checked by one researcher.

### Statistical analysis

Data were extracted and organized in a Microsoft Office Excel^®^ for Mac (version 16.51, Redmond, WA, USA) spreadsheet and analyzed using the Statistical Package for Social Sciences (SPSS for Mac, version 25.0; SPSS Inc., IBM Corp., Armonk, NY, USA). Data analysis included descriptive statistics and absolute and relative frequencies of interest variables. Kolmogorov-Smirnov test was used to assess the normality of variables. Spearman’s correlation was used to assess correlations between variables of interest.

## Results

The search strategy resulted in 448,804 papers. After arranging them in descending order of citations, nine conference papers were excluded. The 100 most-cited papers on Dentistry were cited 101,811 times, including 74 (0.1%) self-citations, ranging between 619 to 2912 citations each.

Table 1 presents the characteristics of the 100 included papers. Their year of publication ranged over six decades. The gender of 13 (3.2%) authors could not be identified, including one (0.2%) lead author, 10 (2.5%) intermediate authors, and two (0.5%) senior authors. For single-authored papers (n=18), the author was considered as lead author. Regardless of authorship role, there were 4.8 male authors for each female one. Among lead authors, there were 11.3 males for each female, and as senior authors, there were 7 males for each female author.

**Table 1:** Frequencies of characteristics of the 100 most-cited papers in Dentistry, according to WoS-CC

Characteristics	n (%)
Decade of publication	100 (100.0)
1960 to 1969	6 (6.0)
1970 to 1979	10 (10.0)
1980 to 1989	18 (18.0)
1990 to 1999	20 (20.0)
2000 to 2009	35 (35.0)
2010 to 2020	11 (11.0)
Number of authors per publication	100 (100.0)
1-2	42 (42.0)
3-4	27 (27.0)
5-6	13 (13.0)
>6	18 (18.0)
Total number of authors (lead, senior, intermediate)	407 (100.0)
Total female authors	68 (16.7)
Total male authors	326 (80.1)
Total not identified	13 (3.2)
Lead author gender	100 (100.0)
Female	8 (8.0)
Male	91 (91.0)
Not identified	1 (1.0)
Intermediate authors' gender	225 (100.0)
Female	50 (22.2)
Male	165 (73.3)
Not identified	10 (4.5)
Senior author gender	82 (100.00)
Female	10 (12.2)
Male	70 (85.4)
Not identified	2 (2.4)
Study design	100 (100.0)
Non systematic Review	41 (41.0)
Laboratory	16 (16.0)
Non randomized Clinical Trial	12 (12.0)
Cross-sectional study	10 (10.0)
Validation study	6 (6.0)
Case‎/series report	5 (5.0)
Cohort study	5 (5.0)
Systematic review	3 (3.0)
Case-control	1 (1.0)
Randomized Clinical Trial	1 (1.0)
Study subject	100 (100.0)
Periodontology	28 (28.0)
Oral and Maxillofacial Surgery	14 (14.0)
Dental Materials	13 (13.0)
Oral Biology	13 (13.0)
Endodontics	10 (10.0)
Implantology	8 (8.0)
Dental Public Health	6 (6.0)
Oral Pathology	4 (4.0)
Orthodontics	2 (2.0)
Oral and Maxillofacial Radiology	1 (1.0)
Paediatric Dentistry	1 (1.0)

Table 2 presents the distribution of the gender of lead, intermediate, and senior authors according to study variables of interest. In the lead authorship analysis, 99 studies were included. Because single-authored papers were considered only in the lead author analysis, the senior authorship analysis included 80 studies. As for the intermediate authors, 215 were included. The independent variable journal of publication was categorized according to the number of publications among the Top 100 most-cited publications in Dentistry (three or more published studies). Turkey and Dubai were considered as part of Asia. Nonsystematic reviews were the most frequent study design among the 100 papers. There was no female lead author of validation, case series, cohort, systematic review, case-control, and randomized clinical trial studies. Senior female authors published nonsystematic reviews (n=7), non-randomized clinical trials (n=1), and validation studies (n=1), only. Female participation as lead and senior authors remained small and relatively stable in all citation density quintiles. Female lead authors ranged from 0 in the second higher quintile to 3 in the lowest one. Among intermediate authors, papers with higher citation density showed higher female participation (n=22). There were more female senior authors in the highest citation count quintile (n=4).

**Table 2:** Distribution of the lead and senior authors by gender from the top 100 most-cited articles in Dentistry (WoS-CC), according to the independent variables (N=100).

Variable/Category		Lead author gender**			Intermediate author gender**			Senior author gender**	
	Total n* (%)	**Female *n* (%)**	**Male *n* (%)**	Total n* (%)	**Female *n* (%)**	**Male *n* (%)**	Total n* (%)	**Female *n* (%)**	**Male *n* (%)**
Study design									
Non Systematic Review	41 (41.0)	3 (7.3)	38 (92.7)	82 (36.4)	21 (26.0)	60 (74.0)	41 (41.0)	7 (24.1)	22 (75.9)
Laboratory study	16 (16.0)	2 (13.3)	13 (86.7)	42 (18.8)	7 (18.0)	32 (82.0)	16 (16.0)	0	13 (100.0)
Non Randomized Clinical Trial	12 (12.0)	2 (16.7)	10 (83.3)	24 (10.7)	8 (34.8)	15 (65.2)	12 (12.0)	1 (9.1)	10 (90.9)
Cross-sectional study	10 (10.0)	1 (10.0)	9 (90.0)	26 (11.7)	6 (25.0)	18 (75.0)	10 (10.0)	0	10 (10.0)
Validation study	6 (6.0)	0	6 (100.0)	10 (4.1)	3 (33.3)	6 (66.7)	6 (6.0)	1 (25.0)	3 (75.0)
Case/series report	5 (5.0)	0	5 (100.0)	5 (2.0)	0	5 (100.0)	5 (5.0)	0	3 (100.0)
Cohort Study	5 (5.0)	0	5 (100.0)	12 (5.4)	3 (25.0)	9 (75.0)	5 (5.0)	0	5 (100.0)
Systematic Review	3 (3.0)	0	3 (100.0)	13 (5.8)	1 (8.3)	11 (91.7)	3 (3.0)	0	3 (100.0)
Case-control study	1 (1.0)	0	1 (100.0)	7 (3.2)	1 (16.7)	5 (83.3)	1 (1.0)	0	1 (100.0)
Randomized Clinical Trial	1 (1.0)	0	1 (100.0)	4 (1.9)	0	4 (100.0)	1 (1.0)	1 (100.0)	0
Citation Density									
913.00 to 76.32	20 (20.0)	2 (10.0)	18 (90.0)	88 (39.1)	22 (26.8)	60 (73.2)	20 (20.0)	4 (23.5)	13 (76.5)
74.93 to 55.13	20 (20.0)	0	20 (100.0)	54 (24.0)	15 (28.3)	38 (71.7)	20 (20.0)	2 (11.8)	15 (88.2)
52.64 to 33.52	20 (20.0)	2 (10.0)	18 (90.0)	49 (21.8)	9 (18.8)	39 (81.2)	20 (20.0)	2 (11.8)	15 (88.2)
31.68 to 21.38	20 (20.0)	1 (5.0)	19 (95.0)	21 (9.3)	2 (10.0)	18 (90.0)	20 (20.0)	1 (6.7)	14 (93.3)
21.11 to 12.40	20 (20.0)	3 (15.8)	16 (84.2)	13 (5.8)	2 (16.7)	10 (83.3)	20 (20.0)	1 (7.1)	13 (92.9)
The time period of publication									
1960 to 1969	6 (6.0)	1 (16.7)	5 (83.3)	2 (0.9)	1 (50.0)	1 (50.0)	5 (6.1)	0	5 (100.0)
1970 to 1979	10 (10.0)	2 (22.2)	7 (77.8)	8 (3.6)	2 (28.6)	5 (71.4)	8 (9.8)	1 (14.2)	6 (85.8)
1980 to 1989	18 (18.0)	0	18 (100.0)	12 (5.3)	0	11 (100.0)	12 (14.6)	2 (18.2)	9 (81.8)
1990 to 1999	20 (20.0)	1 (5.0)	19 (95.0)	47 (20.9)	9 (20.0)	36 (80.0)	18 (21.9)	1 (5.6)	17 (94.4)
2000 to 2009	35 (35.0)	3 (8.6)	32 (91.4)	85 (37.8)	21 (25.0)	63 (75.0)	29 (35.4)	3 (10.3)	26 (89.7)
2010 to 2020	11 (11.0)	1 (9.1)	10 (90.9)	71 (31.5)	17 (25.8)	49 (74.2)	10 (12.2)	3 (30.0)	7 (70.0)
Journal									
J Dent Res	14 (14.0)	1 (7.1)	13 (92.9)	47 (20.9)	9 (20.0)	36 (80.0)	13 (15.9)	2 (15.4)	11 (84.6)
J Periodontol	11 (11.0)	2 (18.2)	9 (81.8)	21 (9.3)	4 (20.0)	16 (80.0)	10 (12.2)	0	10 (100.0)
J Clin Periodontol	9 (9.0)	1 (11.1)	8 (88.9)	18 (8.0)	6 (37.5)	10 (62.5)	8 (9.7)	0	8 (100.0)
Oral Surg Oral Med Oral Pathol Oral Radiol Endod	8 (8.0)	0	7 (100)	16 (7.0)	1 (6.2)	15 (93.8)	6 (7.3)	1 (16.7)	5 (83.3)
Dent Mater	7 (7.0)	1 (14.3)	6 (85.7)	11 (4.9)	4 (36.4)	7 (63.6)	5 (6.1)	2 (40.0)	3 (60.0)
J Oral Maxillofac Surg	7 (7.0)	0	7 (100.0)	13 (5.8)	3 (23.1)	10 (76.9)	4 (4.9)	1(25.0)	3(75.0)
J Endod	5 (5.0)	0	5 (100.0)	9 (4.0)	0	8 (100.0)	4 (4.9)	0	4 (100.0)
Community Dent Oral Epidemiol	3 (3.0)	0	3 (100.0)	5 (2.2)	3 (60.0)	2 (40.0)	1 (1.2)	0	1 (100.0)
J Prosthet Dent	3 (3.0)	1 (33.3)	2 (66.7)	0	0	0	2 (2.4)	0	2 (100.0)
Others (1-2 publications)	33 (33.0)	2 (6.1)	31 (93.9)	85 (37.9)	20 (24.7)	61 (75.3)	29 (35.4)	4 (14.8)	23 (85.2)
Lead author country									
North America	55 (55.0)	7 (13.0)	47 (87.0)	115 (51.2)	28 (25.7)	81 (74.3)	42 (51.2)	4 (9.8)	37 (90.2)
Europe	35 (35.0)	0	35 (100.0)	77 (34.1)	15 (19.7)	61 (80.3)	30 (36.6)	5 (17.2)	24 (82.8)
Asia	8 (8.0)	1 (12.5)	7 (87.5)	26 (11.6)	4 (17.4)	19 (82.6)	8 (9.8)	1 (12.5)	7 (87.5)
Oceania	1 (1.0)	0	1 (100.0)	7 (3.1)	3 (42.9)	4 (57.1)	1 (1.2)	0	1 (100.0)
Latin America	1 (1.0)	0	1 (100.0)	0	0	0	1 (1.2)	0	1 (100.0)
Senior author country									
North America	38 (46.9)	8 (21.1)	30 (78.9)	101 (45.0)	23 (24.0)	73 (76.0)	38 (46.9)	5 (13.2)	33 (86.8)
Europe	37 (45.7)	0	37 (100.0)	96 (42.6)	17 (18.3)	76 (81.7)	37 (45.7)	5 (13.9)	31 (86.1)
Asia	5 (6.2)	0	5 (100.0)	23 (10.2)	7 (33.4)	14 (66.6)	5 (6.2)	0	5 (100.0)
Oceania	1 (1.2)	0	1 (100.0)	5 (2.2)	3 (60.0)	2 (40.0)	1 (1.2)	0	1 (100.0)
Latin America	0	0	0	0	0	0	0	0	0
Senior author gender									
Male	70 (87.5)	7 (10.0)	63 (90.0)	204 (90.7)	45 (23.1)	150 (76.9)			
Female	10 (12.5)	1 (10.0)	9 (90.0)	21 (9.3)	5 (25.0)	15 (75.0)			

*Percentage distribution for the columns; ** Percentage distribution for the rows.

In each decade, female researchers’ presence as lead or senior authors ranged from 0 (1980-1989) to 3 (2000-2009). Female contribution through time, regardless of authorship role, increased along the 20^th^ century, reaching 27 women in the years 2000-2009, and declined in the most recent decade (21 women).

The most frequent journal was the Journal of Dental Research (14 papers), followed by the Journal of Periodontology (11 papers). Of the 55 papers whose lead authors were from North America, seven (13.0%) had a female researcher in the position, while of the eight papers with Asian lead authors, only one had a woman in the position. In Latin America, Europe, and Oceania there was no woman as lead author of any of the most-cited papers. Of the 38 papers whose senior authors were from North America, five (13.2%) had a female researcher in the position. Of the 37 papers with European senior authors, only five (13.9) had a woman in a senior position. In papers from Latin America, Asia, and Oceania no woman was the senior author (Table 2).

Among the 18 lead or senior female authors identified in the 100 most-cited lists, five published up to nine papers each (citations ranged from 983 to 1980), six published more than 100 papers each (citations ranged from 6308 to 20339), and seven published 10 to 86 papers each (citations ranged 646 to 3895) (Table 3). For female authors included in the 100 most-cited lists, the time span between their first and last published paper ranged from 4 to 42 years (mean 27.1 ± 11.9, for lead authors) and 1 to 39 years (mean 25.8 ± 13.6, for senior authors). H factor among female lead and senior authors ranged from 1 to 73 (mean 23.6 + 22.7) (Table 3).

**Table 3:** Female leadership in the 100 most-cited papers in Dentistry (WoS-CC)

Author	Country	Total citations	Total Papers	H factor	i500	Lead authorship (%)	Last authorship (%)	Year of the first paper	Year of last paper	Study subject
Lead										
Tanner ACR	United States	8848	146	46	2	36	18	1978	2020	Periodontology
Grossi SG	United States	6308	116	38	2	20	4	1981	2011	Periodontology
Denry I	United States	3895	86	23	2	63	13	1985	2021	Dental Materials
Guo S	China	2156	3	2	1	67	0	2009	2013	Oral Biology
Haraszthy VI	United States	1646	43	15	1	42	12	1990	2019	Periodontology
Humphrey SP	United States	1075	4	2	1	50	10	1980	2002	Oral Biology
Gold OG	United States	983	8	4	1	75	0	1973	1992	Oral Biology
Quigley GA	United States	924	12	7	1	17	67	1961	1996	Periodontology
Senior										
Haffajee AD	United States	20339	245	73	5	26	16	1978	2015	Periodontology
Wennerberg A	Sweden	14338	264	60	2	11	51	1991	2022	Implantology
DiPietro LA	United States	13390	205	48	5	14	55	1982	2021	Oral Biology
Van Landuyt K	Belgium	11977	153	49	5	15	15	2003	2022	Dental Materials
O'Ryan F	United States	3114	48	19	2	44	21	1980	2017	Oral and Maxillofacial Surgery
Bay I	Denmark	1980	9	5	1	44	44	1973	2010	Periodontology
Georgeff KR	United States	1727	1	1	1	0	100	1998	1998	Oral and Maxillofacial Surgery
Kao EC	United States	1273	53	15	1	53	15	1986	2019	Dental Materials
Sardo-Infirri J	France	1055	10	7	1	20	60	1977	1987	Dental Public Health
Dorigo ES	Italy	646	13	10	0	0	8	1997	2011	Dental Materials

**Figure 1:**
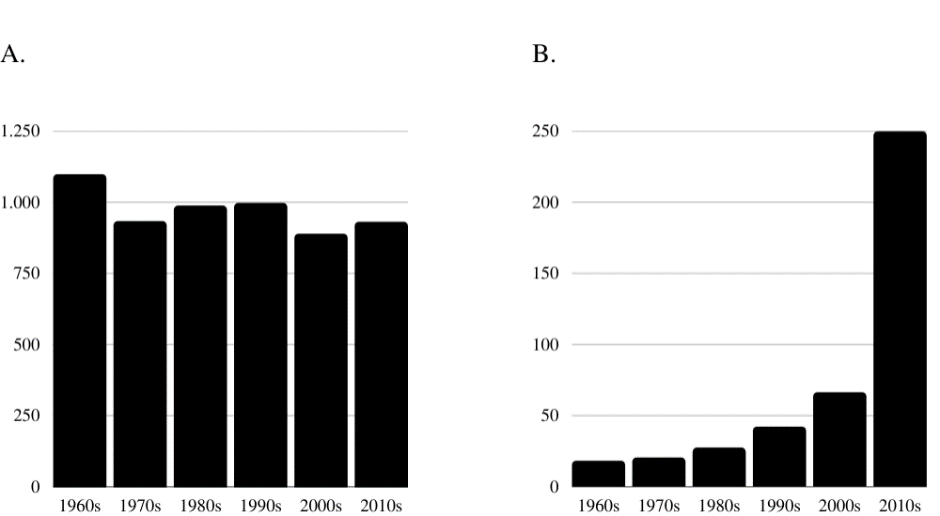
Mean Citation indicators of each article by decade. A) Mean citation count; B) mean citation density.

The mean citation count remained relatively stable over time, while citation density increased as decades passed (Figure 1). Years since paper publication ranged from 1962 to 2020 (median=1998). The citation density of the most-cited papers ranged from 12.40 to 913.00. There was a positive, moderate correlation between citation density and citation count (Spearman’s ρ=0.438; p<0.01), being citation density dependent on the citation count of each article. Furthermore, there was a very high correlation between publication year and citation density (Spearman’s ρ=0.847; p<0.01), showing that citation density is highly influenced by the time in which the paper has been published. (Table 4).

**Table 4:** Spearman’s correlation between variables citation density, citation count, and publication year.

	Citation density	Citation count	Publication year
Citation density	-		
Citation count	0.438**	-	
Publication year	0.847**	-0.024**	-

**p<0.01

## Discussion

This bibliometric study assessed the participation of women as lead, intermediate, and senior authors of the 100 most-cited papers in Dentistry, as a measure of female leadership in dental research from a historical perspective, as papers were published across 60 years. Findings from this study show the underrepresentation of women in the authorship of top-cited papers. In Dentistry, female representation has been reported to increase in the last decades across different domains. Women make up most Dental School graduates in Canada, France, and the United Kingdom. Women also make most registered dentists in Brazil, Chile, and India and most dental researchers in Brazil^5^ ^,^ ^15^. However, American studies show that women are underrepresented as leaders in dental research, as members of editorial boards in the role of chief and associate editors^7^, and as dean in Schools of Dentistry^6^.

In this study, data collected on lead and senior female authors highlight their contributions to dental science as described through the citation counts of their papers. The h index is used to measure the academic contribution of researchers and provides a measure of both quantity and quality, as it combines the number of publications and citations of one author^16^. However, the index may benefit senior researchers and those in later career stages, whether they are still actively publishing or not^16^. Senior authors may play an additional role in mentoring junior researchers.

The findings of this study show that although disproportionally small, female participation in paper authorship increased as time passed. Dr. Brooks, one of the female authors of the papers included in the most-cited list, noted the gender inequality and challenges faced by female faculty and the increase in representation through time:

"…*at the time that I started here [at the University of Michigan], there weren’t very many women around… and some of the faculty didn’t quite know what to do with me... We were a real novelty. [Now,] nobody cares whether you’re a woman or a man anymore*”^17^.

The trend for more women as authors of dental papers echoes the recent increase in their participation in dental academia. The number of female dental researchers increased between 1996-2000 and 2011-2015 in several European, North, and Latin American countries^15^. However, the literature states that there has been limited advance and important challenges to achieve greater female representation in leadership roles^6^ ^,^ ^7^, as seen in the 100 most-cited papers in dental research from a historical perspective.

There was great global disparity between different continents, as represented by the country of the author’s affiliation. Most papers included in the present study were authored by researchers affiliated with institutions located in the global north, regardless of gender or authorship role. From a historical point of view, the results call for greater representation of both male and female authors affiliated with institutions from the southern hemisphere. However, it is noteworthy that current data from countries such as Brazil show great participation in Dental Research worldwide^18^. In this way, it is possible that in the present/near future authors of top-cited papers will be less homogeneous regarding the author's affiliation.

This study found differences in female authorship in dental research across continents. It reinforces the marked gender inequality also observed in a previous study among authors originating from North America, Europe, and Asia^8^. A recent study reported that the proportional participation of women among dental researchers reaches 35% in the United States, and 33% in the European Union. In Japan, only 25% of dental researchers are women^5^. Counterintuitive as it may seem, although female researchers are in smaller figures than their male colleagues, in some countries the proportion of registered female dentists is known to be almost equal to registered male dentists. Women are 49% of dentists in the United States, 52% in Canada, 56% in the United Kingdom, 55% in France, and 55% in Germany^5^. Women also make up most (51%) of last year's students in Dental Schools in the United States^19^.

Despite being heavily underrepresented in the authorship of the top 100 most-cited dental papers; authorship is one way of evaluating female leadership in dental science. Other proxy domains can also identify women who challenge the status quo of gender dominance. They managed to excel in their endeavors and many have also stood the test of time^20^. Female dental researchers have received awards^20^, invitations to speak at conferences^21^, and received millions of dollars in extramural research grants^22^.

In academic Dentistry, the vital role of mentorship for future academics is undeniable. In addition, the role of women scientists as mentors who offer support and guidance is considered a highly valuable contribution to the successful careers of other women in science. Beyond inspiring role models, recent evidence shows women leaders are paramount in the fight against gender inequalities. In academic publishing, female senior authors have been shown to increase the participation of female lead authors^8^; while in dental education, it has been reported that schools with female deans present a higher number of women in other jobs^6^.

Citation counts are frequently used as a measure of scientific impact. However, they may be susceptible to temporal effect, as counts tend to grow in number as time passes and papers published earlier may have more citations^23^. On the other hand, a recently published and highly cited paper reflects a professional interest in new trends and investigations: indeed, two of the most-cited papers were published in 2020 reporting findings on the emergent coronavirus disease 2019^24^. In this study, there was a significant but weak negative correlation between citation count and year of publication. Citation density may be used as a complementary metric to citation count. In the present study, while there was a relatively stable value in the mean citation count by each article per decade, there was a sheer increase in mean citation density in most recent decades, similar to what has been documented in the medical field^25^.

Some 58 years separate the earliest and the latest selected paper. Given such a time span, gender analysis of authorship might have led to an overrepresentation of men. However, the author´s gender distribution according to the paper's citation density shows stable female participation as lead or senior authors.

It must be noted that gender is a non-binary, complex, and socially constructed concept. However, in this paper, gender was assigned in binary categories, male and female. In addition, according to an intersectionality framework, other identities, such as race, sexuality, ethnicity, and economic background might interact with gender, shaping one’s experience of oppression and creating unequal opportunities for female leadership^26^. Future studies should focus on evaluating leadership in Dentistry using intersectional lenses. It should be noted that this paper assessed female representation as authors of the 100-most-cited articles in dental research, and therefore differences across subgroups should interpreted with caution - future studies should expand this analysis to a larger sample of papers. In addition, the search was carried out in 2021 and it is possible that results might have been updated and more studies should be encouraged.

“Feminism is a movement to end sexism, sexist exploitation, and oppression” ^(^ ^27^. It urges all members of society to “let go of sexist thought and action”. It recognizes the role each of us plays in perpetuating systems of oppression. In all fields of science, the underrepresentation of women stems from different barriers, originating from structural, organizational, systemic, institutional, social, and cultural sources ^1^. Still, to break science free of sexism, it is paramount that the essential role of socially constructed individual bias and pervasive negative stereotypes are addressed.

Women were found to be largely underrepresented as leaders of the top cited papers in Dentistry and there is a need for action. It is important to stimulate female leadership and contributions to shape the future of Dental Research and Education by overcoming long-standing beliefs, societal norms, stereotypes, and biases that may lead to the unequal participation of women in dental research.
